# Supplementary material for: STING agonist-loaded, CD47/PD-L1-targeting nanoparticles potentiate antitumor immunity and radiotherapy for glioblastoma
Source: Nat Commun. 2023 Mar 23;14:1610. doi: 10.1038/s41467-023-37328-9 (PMC10036562; doi:10.1038/s41467-023-37328-9)
Supplement: Supplementary file 1 — Supplementary information [file 41467_2023_37328_MOESM1_ESM.pdf]

STING agonist-loaded, CD47/PD-L1-targeting nanoparticles potentiate antitumor immunity and radiotherapy for glioblastoma

Peng Zhang[1, *], Aida Rashidi[1], Junfei Zhao[2,3], Caylee Silvers[1], Hanxiang Wang[1], Brandyn Castro[1], Abby Ellingwood[1], Yu Han[1], Aurora Lopez-Rosas[1], Markella Zannikou[1], Crismita Dmello[1], Rebecca Levine[1], Ting Xiao[1], Alex Cordero[1], Adam M. Sonabend[1], Irina V. Balyasnikova[1], Catalina Lee-Chang[1], Jason Miska[1, *], Maciej S. Lesniak[1, *]

[1]Department of Neurological Surgery, Lou and Jean Malnati Brain Tumor Institute, Northwestern University Feinberg School of Medicine, Chicago, IL, USA

[2]Program for Mathematical Genomics, Department of Systems Biology, Columbia University, New York, NY, USA

[3]Department of Biomedical Informatics, Columbia University, New York, NY, USA

*Correspondence to:

Peng Zhang, peng@northwestern.edu

Jason Miska, jason.miska@northwestern.edu

Maciej S. Lesniak, maciej.lesniak@northwestern.edu

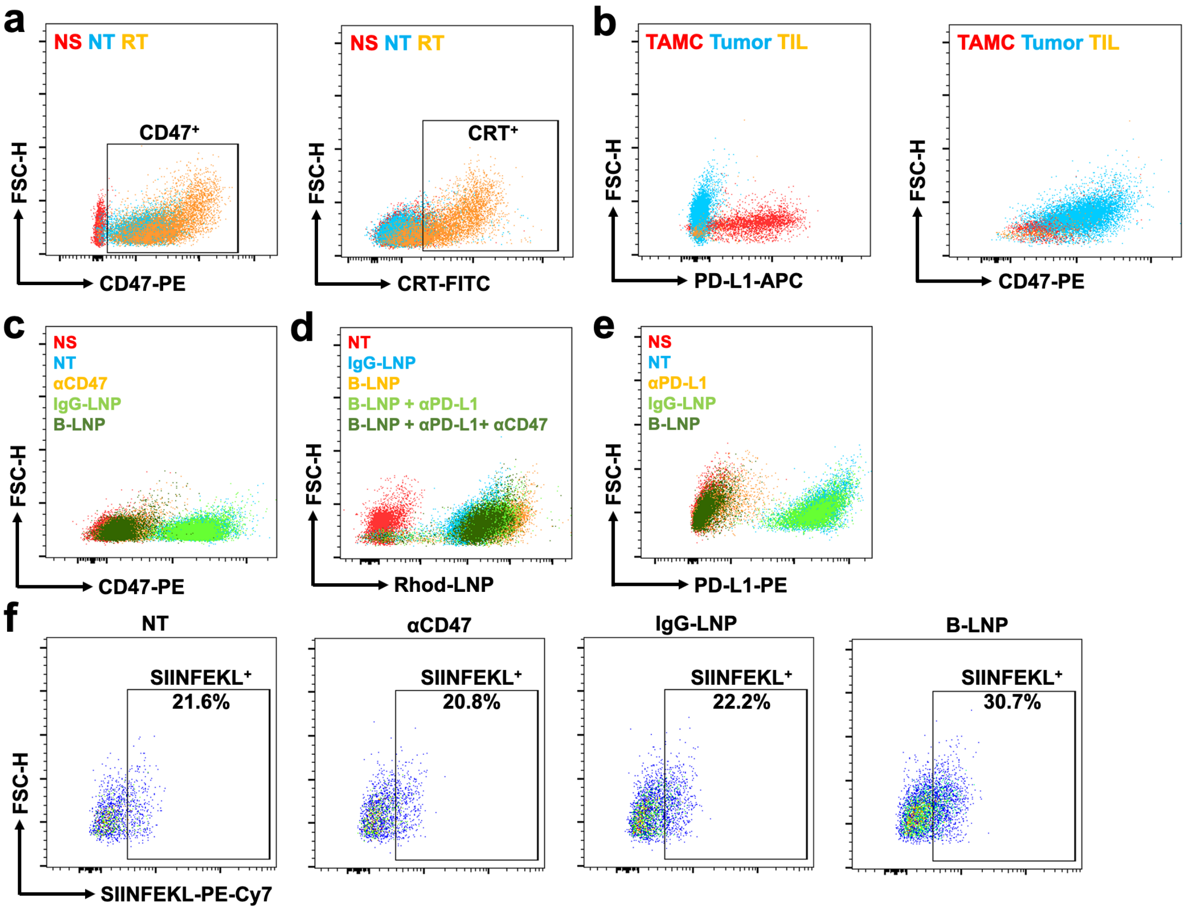

Supplementary Fig. 1 B-LNP targets CD47/PD-L1 checkpoint molecules and promotes TAMC presentation of tumor-associated antigen. a, Representative flow cytometric analysis of radiotherapy (RT)-induced overexpression of CD47 and calreticulin (CRT) in CT-2A glioma cells as quantified in **Fig. 2a-b**. **b,** Representative flow cytometric analysis of PD-L1 and CD47 expression in different subsets of cells in CT-2A-bearing brains 72 h after brain-focused radiotherapy as quantified in **Fig. 2d**. **c,** Representative flow cytometric analysis of CD47 expression in CT-2A cells treated with αCD47 antibody at 25 µg/ml as quantified in **Fig. 2e**. **d,** Representative flow cytometric analysis of binding efficiency of Rhod-tagged B-LNP to CT-2A-associated TAMCs as quantified in **Fig. 2f**. **e,** Representative flow cytometric analysis of PD-L1 expression in CT-2A-associated TAMCs treated with αPD-L1 antibody at 25 µg/ml as quantified in **Fig. 2g**. **f,** Representative flow cytometric analysis of $OVA_{257-264}$ (SIINFEKL) peptide bound to H-$2K^b$ in TAMCs 24 h after co-cultured with CT-2A-OVA as quantified in **Fig. 2m**. NT, non-treated; NS, unstained control; RT, radiotherapy; TAMC, tumor-associated myeloid cell; TIL, tumor-infiltrating lymphocyte.

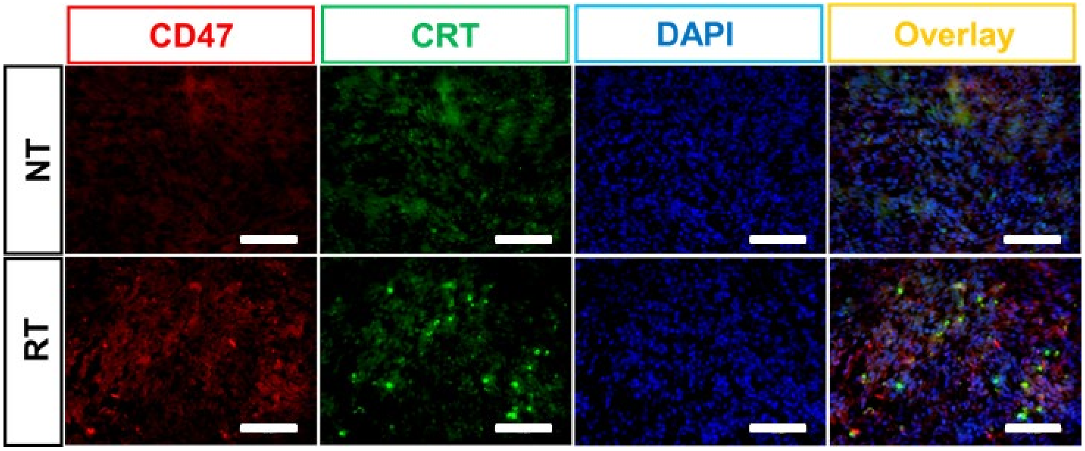

Supplementary Fig. 2 Irradiation triggers upregulation of CD47 and calreticulin in CT-2A brain tumors. C57 mice received intracranial implantation of 7.5 × 10^4 CT-2A cells were treated with brain-focused radiotherapy (RT, 3 Gy × 3) starting on the seventh day post-tumor implantation. Expression of CD47 and CRT in brain tumors 72 h post-RT was evaluated by immunofluorescence staining using Alexa Fluor 488 anti-mouse/rat/human calreticulin (CRT) and Alexa Fluor 647 anti-mouse CD47 at 1:100 dilution. NT, non-treated. The experiment was carried out independently twice. Scale bar, 100 µm.

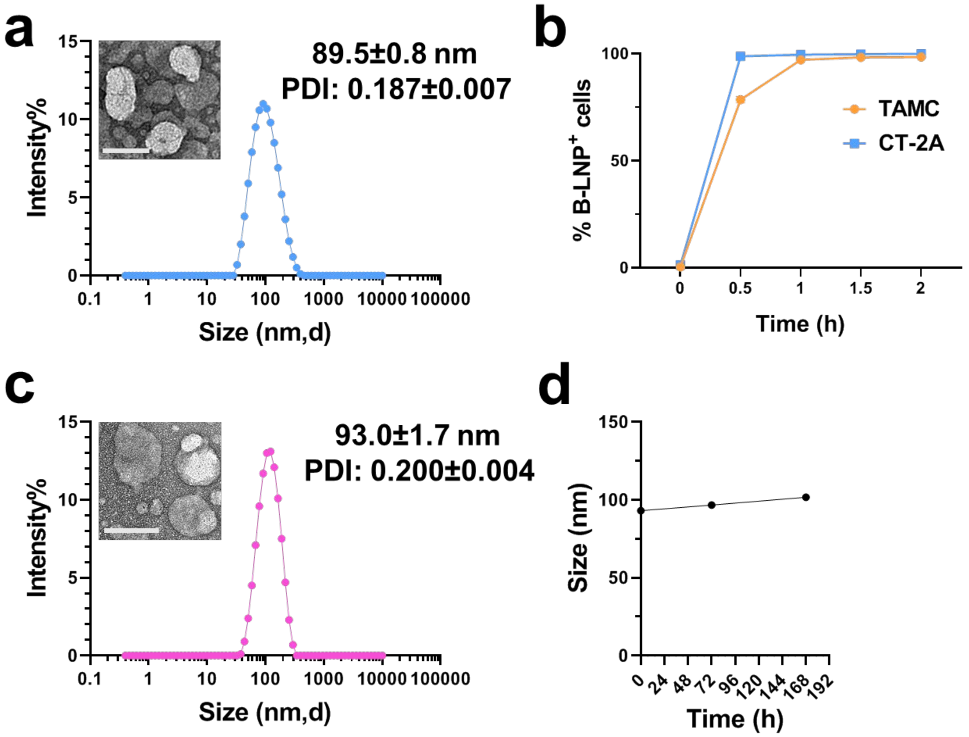

Supplementary Fig. 3 Characterization of B-LNP and B-LNP/diABZI. Particle size distribution of B-LNP (**a**) and B-LNP/diABZI (**c**) was determined by dynamic light scattering (DLS) using a zetasizer. Inserts: transmission electron microscope (TEM) imaging was performed by negative staining using uranyl acetate. The experiment was carried out independently twice. Scale bar, 100 nm. **b**, Binding kinetics of B-LNP to tumor cells and TAMCs were determined by incubating CT-2A and TAMC with Rhod-labeled B-LNP at 4 °C. % B-LNP^{+} CT-2A or TAMC at predetermined time intervals was measured by flow cytometry. **d**, Formulation stability of B-LNP/diABZI was determined by its storage stability at 4 °C as shown by the change of particle size. Source data are provided as a Source Data file.

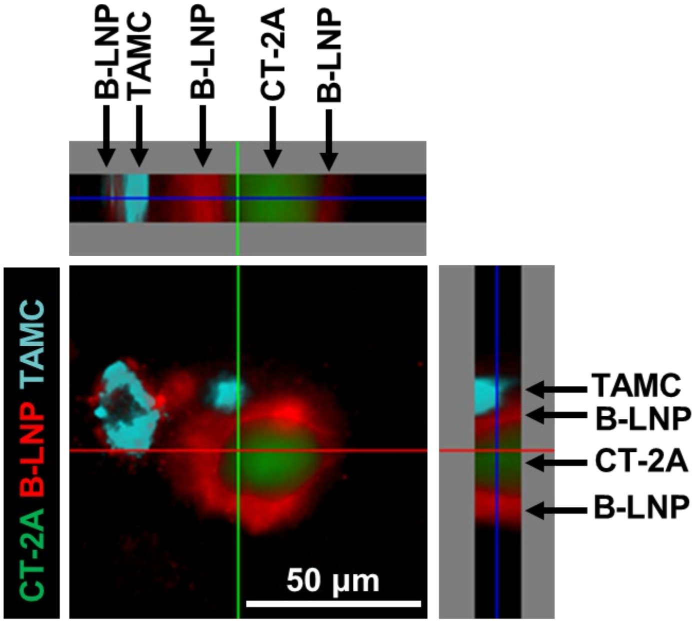

Supplementary Fig. 4 Z-stack imaging of cellular distribution of B-LNP. A co-culture of CT-2A-GFP (green) and TAMC (cyan) was treated with Rhod-tagged B-LNP (red) for 1 h. Z-stack imaging was performed using a Leica DMi8 microscope. The experiment was carried out independently three times.

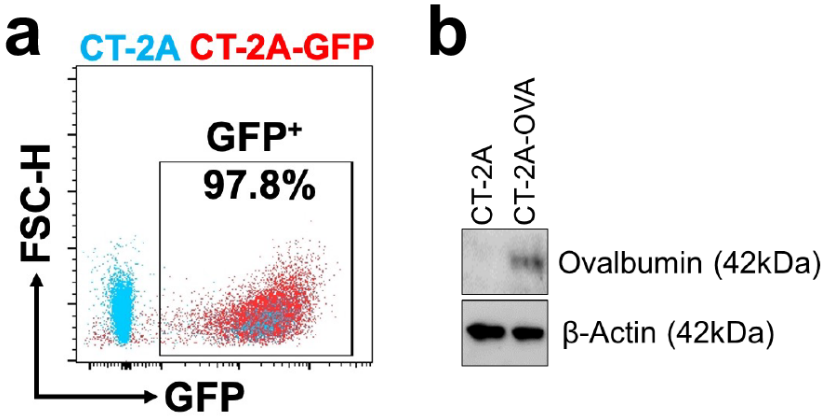

Supplementary Fig. 5 Characterization of genetically modified CT-2A cells. a, Flow cytometric analysis of GFP expression in CT-2A-GFP cells. **b,** Western blot of OVA expression in CT-2A-OVA cells. The experiment was carried out independently three times. Uncropped and unprocessed scan of the blots with molecular markers are provided as a Source Data file.

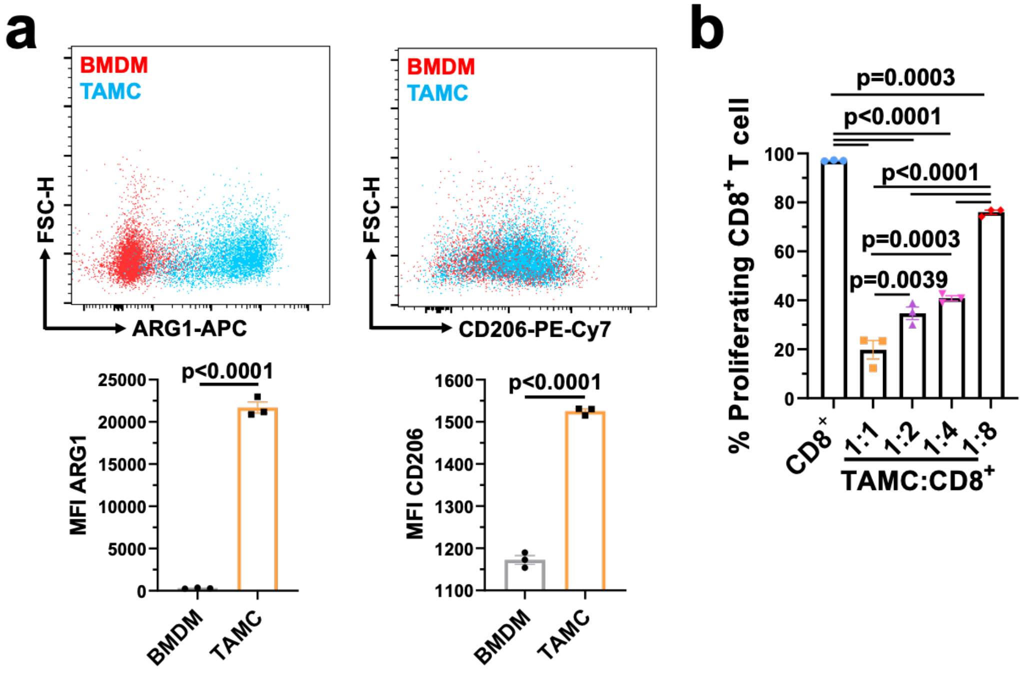

Supplementary Fig. 6 In vitro generated TAMCs demonstrate an immunosuppressive phenotype. Immunosuppressive phenotype of TAMCs was determined by high expression of ARG1 and CD206 (**a**) and inhibitory effect on the proliferation of $CD8^+$ T cells in a co-culture of TAMCs + CellTrace Violet-stained $CD8^+$ T cells at various ratios for 72 h (**b**). BMDM, bone marrow-derived macrophage. n = 3 biological replicates. Statistics were determined by two-sided Student's t-test (in **a**) or one-way ANOVA with Tukey's multiple comparisons test (in **b**); data are presented as mean values +/- SEM. Source data are provided as a Source Data file.

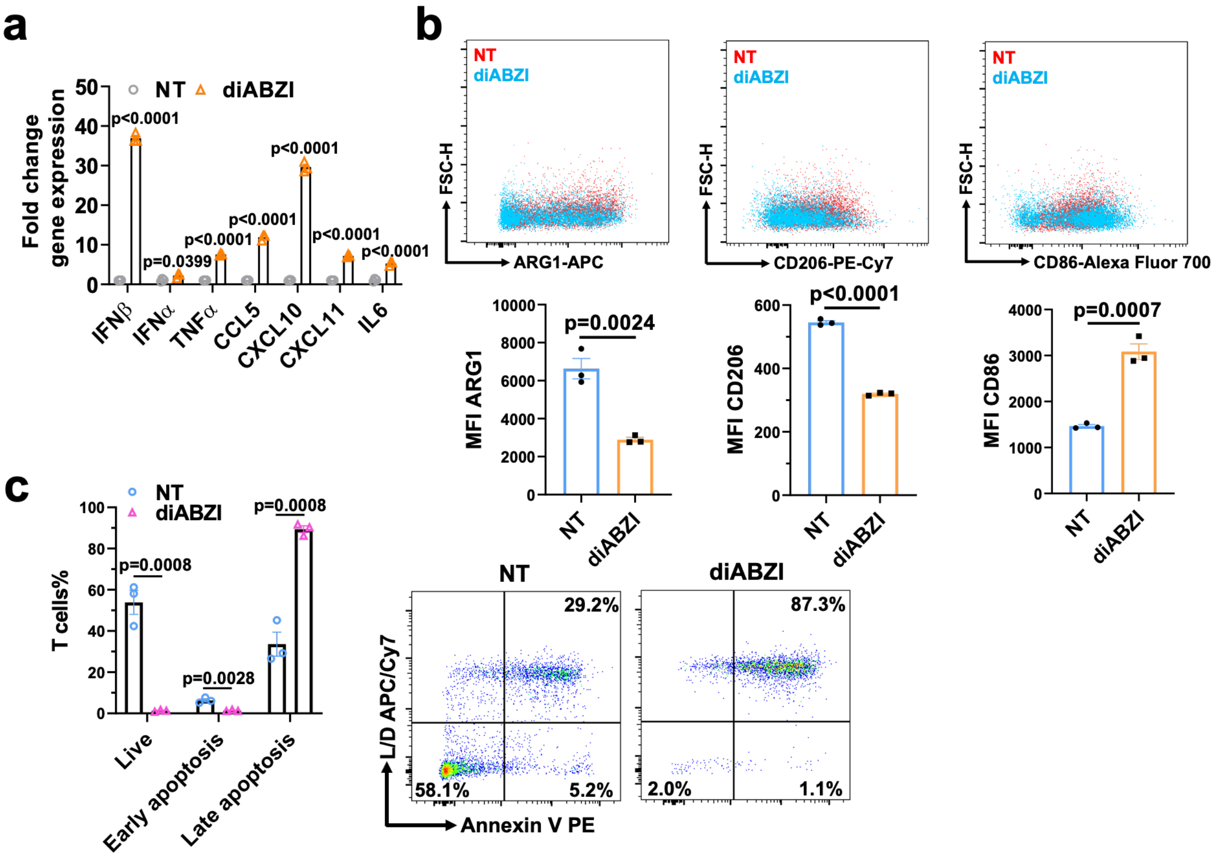

Supplementary Fig. 7 diABZI treatment reprograms TAMC to a pro-inflammatory phenotype but causes cytotoxicity to T cells. a-b, diABZI-treated TAMCs demonstrated a pro-inflammatory phenotype as determined by the expression of a panel of pro-inflammatory cytokines measured by qPCR 6 h post-treatment (**a**) and altered expression of ARG1, CD206, and CD86 measured by flow cytometry 24 h post-treatment (**b**). **c**, Cytotoxicity of diABZI to T cells was tested by Annexin V staining using flow cytometry 48 h after the treatment with diABZI at 200 nM. NT, non-treated. n = 3 biological replicates; statistics were determined by two-sided Student's t-test; data are presented as mean values +/- SEM. Source data are provided as a Source Data file.

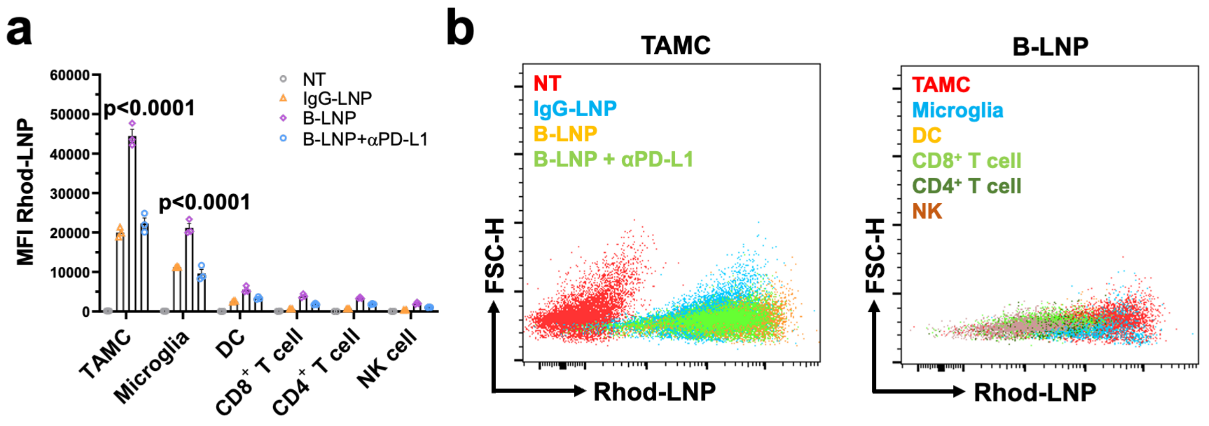

Supplementary Fig. 8 B-LNP demonstrates high binding affinity to the myeloid compartment in murine gliomas. Flow cytometric analysis of binding efficiency of Rhod-tagged B-LNP in subsets of glioma-infiltrating immune cells as represented by mean fluorescence intensity (MFI) (**a**) and representative dot plots (**b**). n = 3 biological replicates; statistics were determined by two-way ANOVA with Tukey's multiple comparisons test; data are presented as mean values +/- SEM. Source data are provided as a Source Data file.

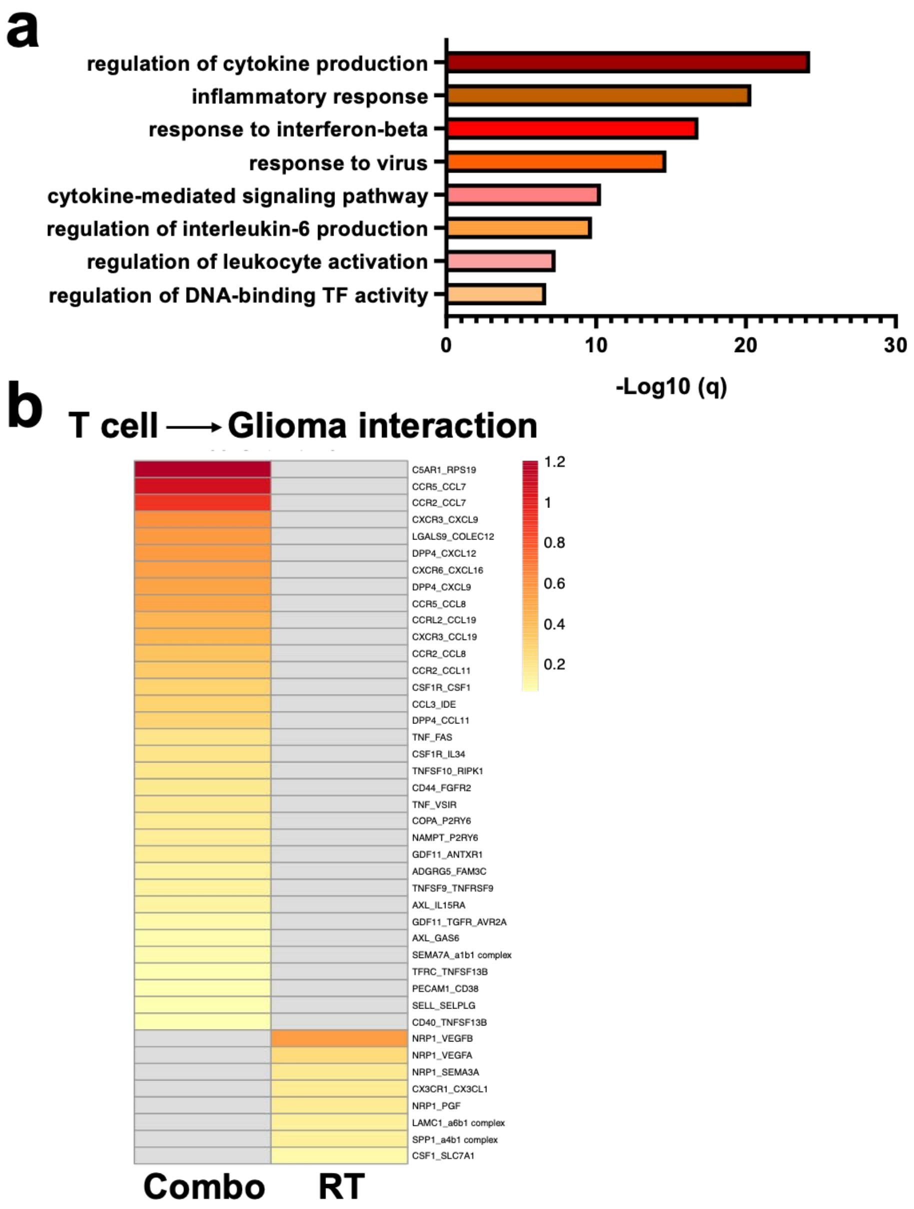

Supplementary Fig. 9 Single-cell RNA sequencing analysis of murine gliomas. Brain tumors were collected from CT-2A-bearing C57 mice received radiotherapy (RT) or RT + B-LNP/diABZI combination therapy (referred to as Combo). **a**, Top enriched pathways in TAMC sub-cluster 1 (as demonstrated in **Fig. 3c**) through GO enrichment analysis. **b**, T cell to GBM cell interaction analysis. Source data are provided as a Source Data file.

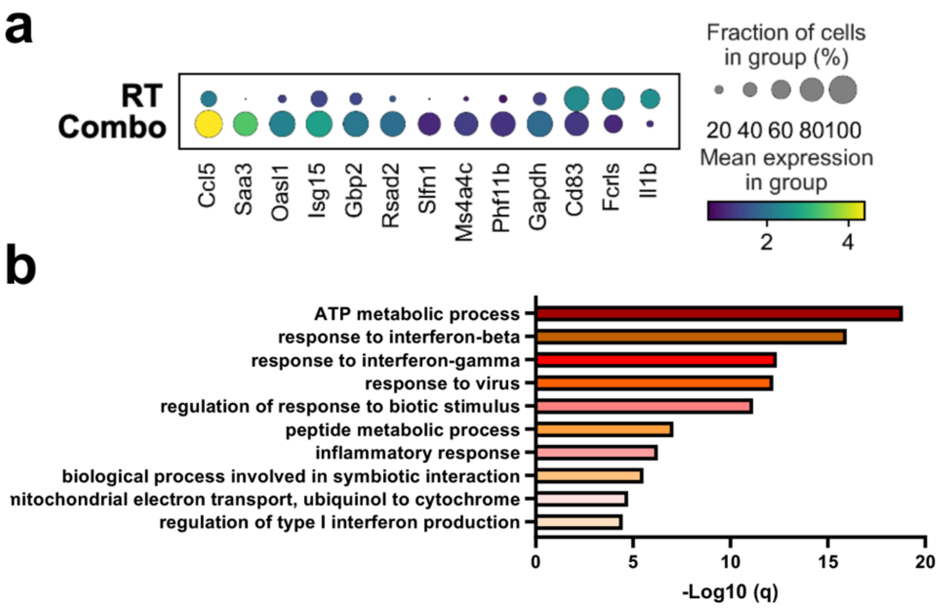

Supplementary Fig. 10 Single-cell RNA sequencing analysis of microglia in murine gliomas. Brain tumors were collected from CT-2A-bearing C57 mice received radiotherapy (RT) or RT + B-LNP/diABZI combination therapy (referred to as Combo). **a,** Unbiased expression analysis indicating top upregulated and downregulated genes in microglia post-Combo treatment as compared to RT alone. **b,** Top enriched pathways in microglia through GO enrichment analysis. Source data are provided as a Source Data file.

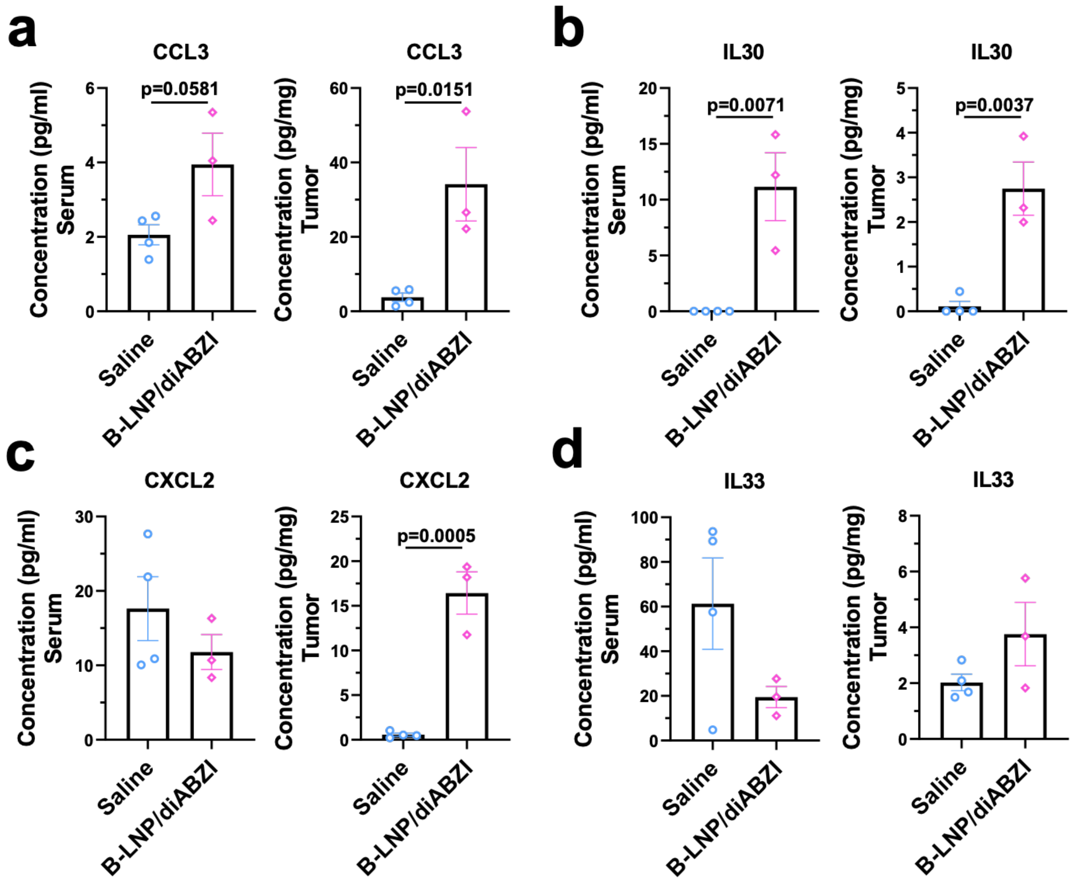

Supplementary Fig. 11 MSD multiplex cytokine analysis. CT-2A-bearing mice were treated by radiotherapy + saline or B-LNP/diABZI (0.25 mg/kg diABZI). A panel of cytokines CCL3 (**a**), IL30 (**b**), CXCL2 (**c**), and IL33 (**d**) in serum and the brain tumor milieu were measured using an MSD V-PLEX Cytokine Panel 1 Mouse Kit (n = 3 or 4 mice/group). IL9, IL15, and IL17 were undetectable in the samples. Statistics were determined by two-sided Student's t-test. Data are presented as mean values +/- SEM. Source data are provided as a Source Data file.

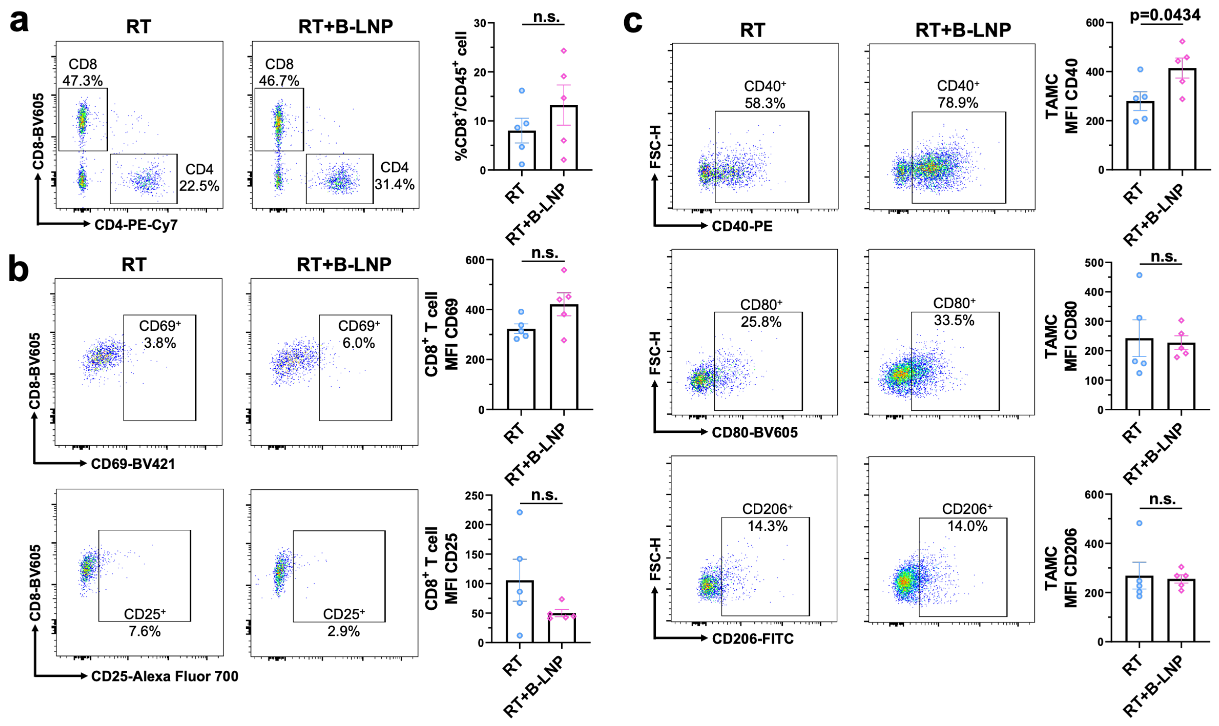

Supplementary Fig. 12 Flow cytometric analysis of the effects of drug-free B-LNP on tumor-infiltrating immune cells. Freshly dissected brains from CT-2A-bearing mice treated with radiotherapy (RT) +/- B-LNP were analyzed by flow cytometry for $CD8^+$ T cell infiltration (**a**) and activation (**b**), and TAMC phenotype (**c**). n = 5 mice. Statistics were determined by two-sided Student's t-test; data are presented as mean values +/- SEM. Source data are provided as a Source Data file.

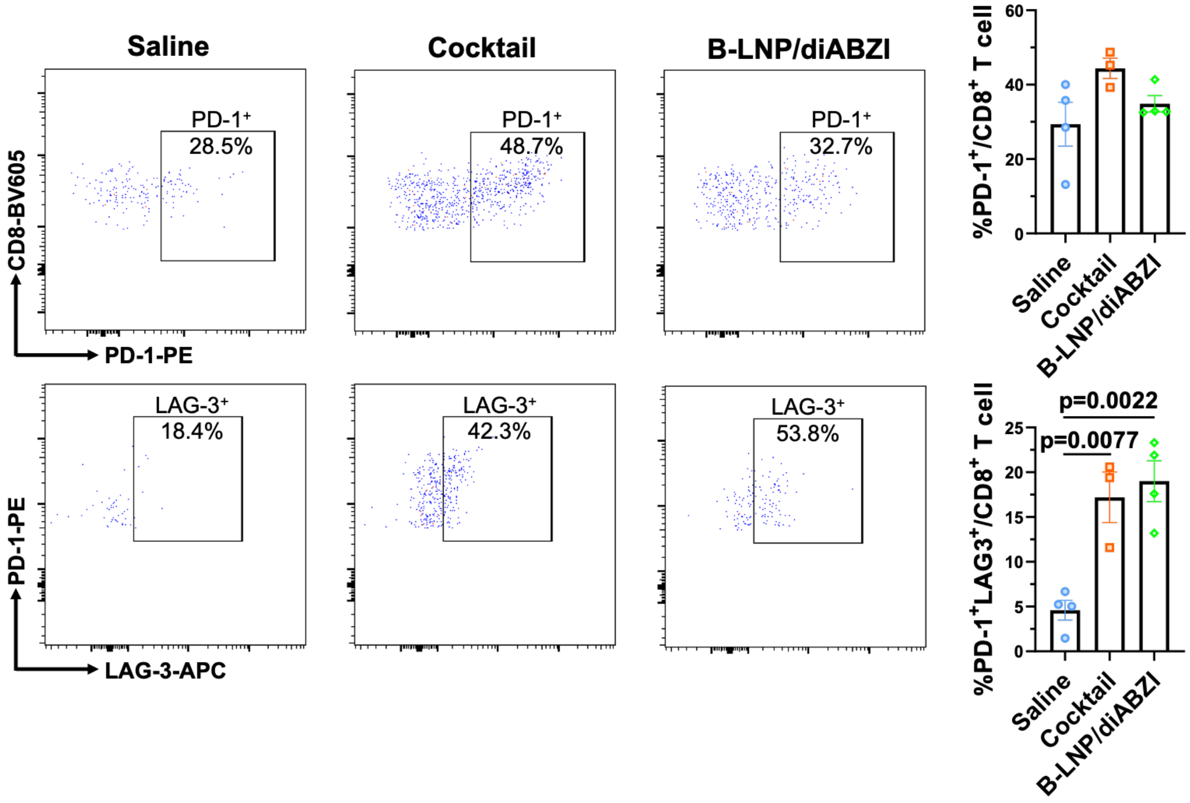

Supplementary Fig. 13 Flow cytometric analysis of CD8^{+} T cell exhaustion. CT-2A-bearing mice were treated with radiotherapy + saline, a combination of free diABZI + αPD-L1 + αCD47 (Cocktail), or B-LNP/diABZI (0.25 mg/kg diABZI). T cells were analyzed by flow cytometry in tumor-bearing brains (n = 3 or 4 mice/group). Statistics were determined by two-sided one-way ANOVA with Tukey's multiple comparisons test; data are presented as mean values +/- SEM. Source data are provided as a Source Data file.

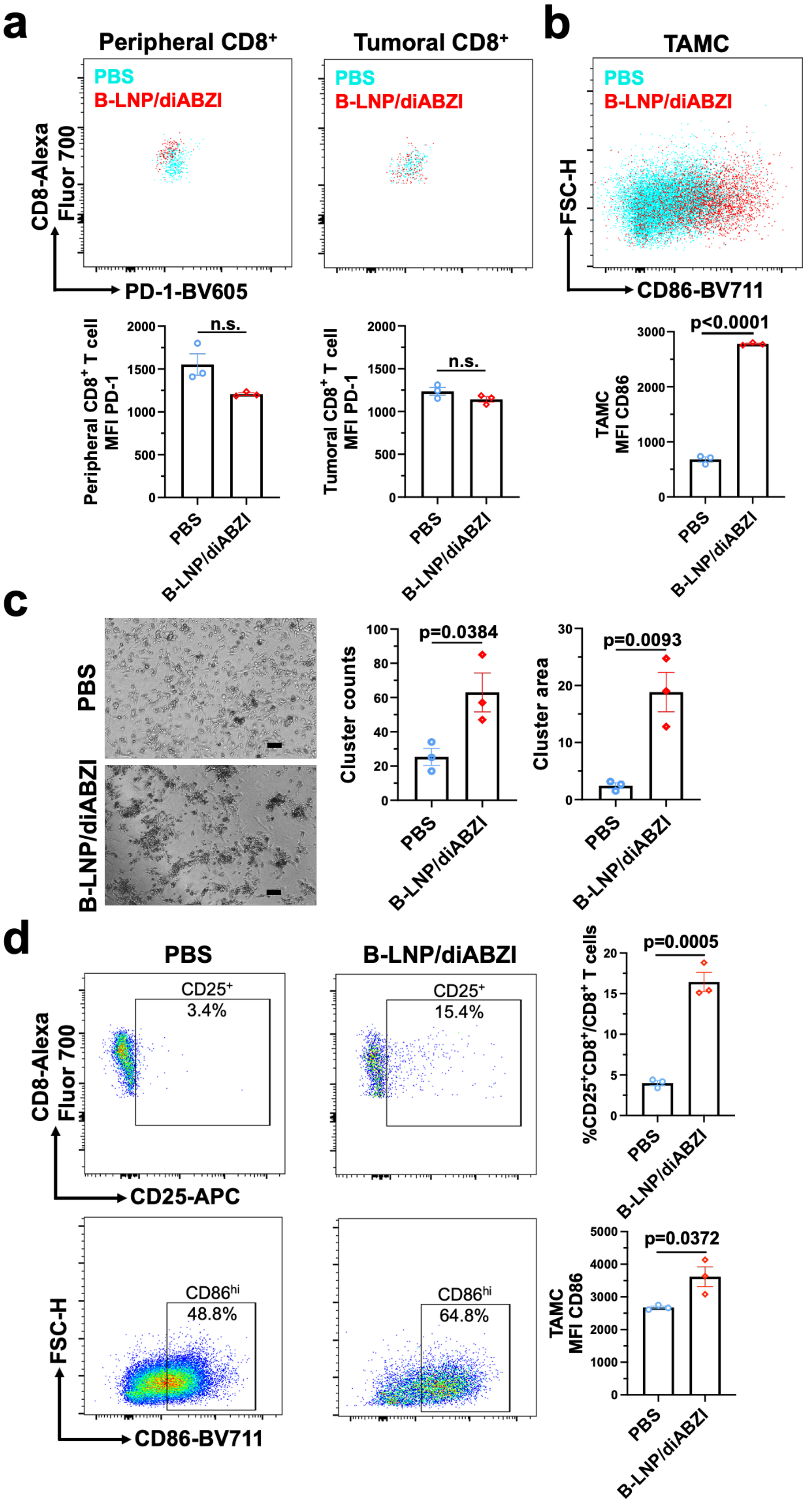
a
Peripheral CD8⁺
Tumoral CD8⁺
PBS
B-LNP/diABZI
CD8-Alexa Fluor 700
PD-1-BV605
Peripheral CD8⁺ T cell MFI PD-1
Tumoral CD8⁺ T cell MFI PD-1
n.s.
b
TAMC
FSC-H
CD86-BV711
TAMC MFI CD86
p<0.0001
c
Cluster counts
Cluster area
p=0.0384
p=0.0093
d
CD25⁺
3.4%
15.4%
CD25-APC
CD86hi
48.8%
64.8%
%CD25⁺CD8⁺/CD8⁺ T cells
p=0.0005
p=0.0372

Supplementary Fig. 14 B-LNP/diABZI induces activation of GBM patient-derived immune cells collected from clinical specimens. a-c, GBM case NU02747. Ex vivo samples were treated with PBS or anti-human CD47/PD-L1 functionalized B-LNP/diABZI at diABZI concentration of 100 nM. **a**, Flow cytometric analysis of PD-1 expression in peripheral and tumoral $CD8^+$ T cells. **b**, Flow cytometric analysis of CD86 expression in TAMCs. **c**, Nanoparticle treatment induced cell-cell clustering interaction in patient-derived immune cells. Scale bar, 60 μm. **d,** GBM case NU03136. Flow cytometric analysis of CD25 expression in $CD8^+$ T cells and CD86 expression in TAMCs. n = 3 independent formulations. Statistics were determined by two-sided Student's t-test; data are presented as mean values +/- SEM. Source data are provided as a Source Data file.

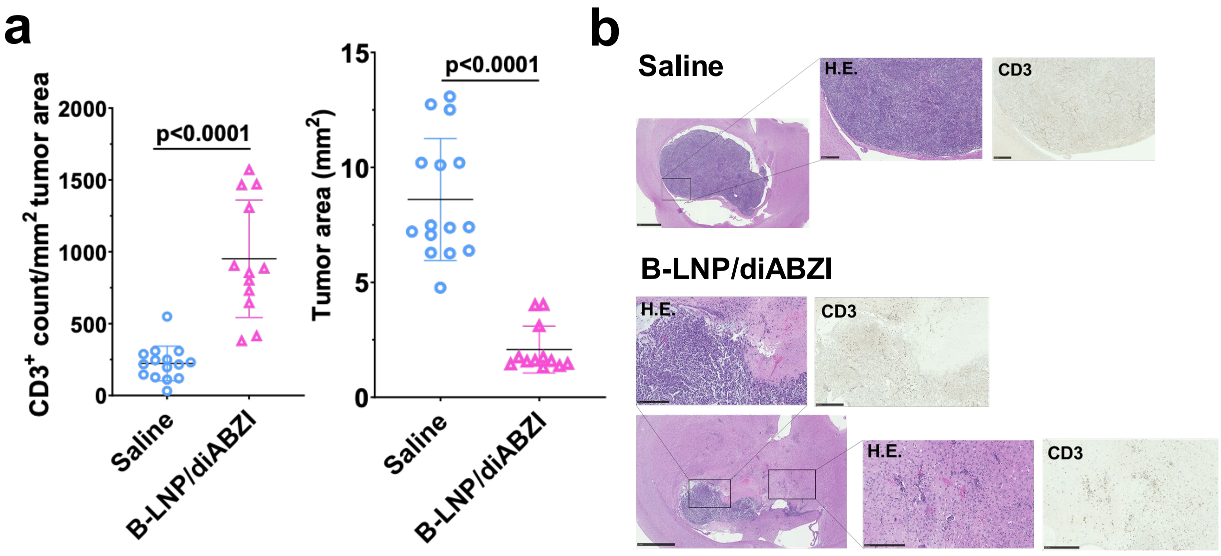

Supplementary Fig. 15 B-LNP/diABZI enhances brain tumor migration of CAR T cells. Rag1$^{-/-}$ mice injected with 2×10^5 CT-2A-IL13Ra2 cells were treated with radiotherapy (RT, 3 Gy × 3) + saline or B-LNP/diABZI treatment (0.25 mg diABZI per kg) through intracranial cannula. 24 hours after nanoparticle treatment, IL13Ra2.CAR T cells were delivered via intravenous route at 1×10^7 cells per mouse. Seventy-two hours later, brain tissues were collected and stained with H&E or anti-CD3 antibody. Quantification (**a**) and representative images (**b**) of H&E and CD3 staining of tumor-bearing brains. Scale bar, 1 mm; insert scale bar, 250 µm. Saline, n = 15 (5 mice, 3 tissue sections each animal; B-LNP/diABZI, n = 12 (4 mice, 3 tissue sections each animal). Statistics were determined by two-sided Student's t-test; data are presented as mean values +/- SEM. Source data are provided as a Source Data file.

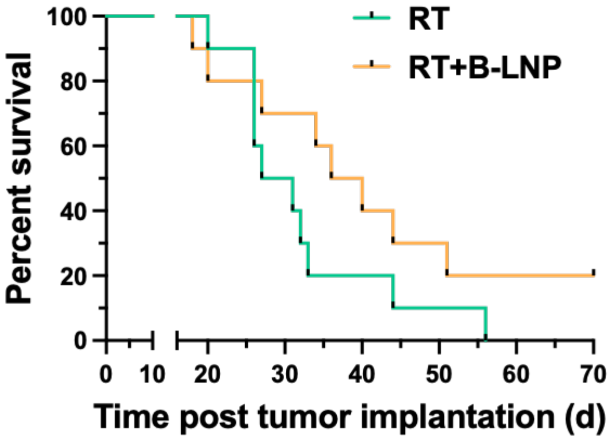

Supplementary Fig. 16 B-LNP treatment moderately improves antitumor effects of radiotherapy in glioma-bearing mice. Survival curves of C57 mice received intracranial implantation of 5×10^4 CT-2A cells, radiotherapy (RT, 3 Gy × 3), and two administrations of saline or drug-free B-LNP through implanted cannula. n = 10 mice per group. No statistical significance as determined by Renyi's test for groups with crossing hazards. Source data are provided as a Source Data file.

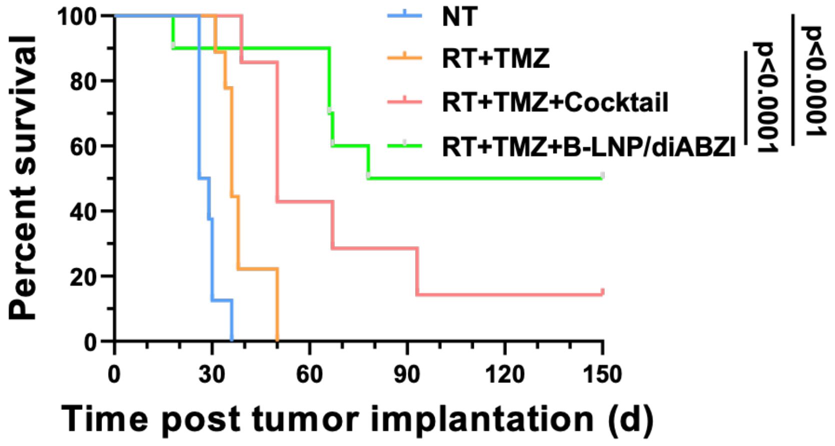

Supplementary Fig. 17 B-LNP/diABZI treatment improves the antitumor effects of radiotherapy plus concomitant adjuvant temozolomide in glioma-bearing mice. Survival curves of C57 mice received intracranial implantation of 5 × 10^4 CT-2A cells and two administrations of saline, B-LNP/diABZI (0.25 mg/kg diABZI), or a combination of free diABZI and therapeutic antibodies (αPD-L1 + αCD47) (referred to as Cocktail) through implanted cannula. Selected groups of mice also received radiotherapy (RT, 3 Gy × 3) plus temozolomide (TMZ) through intraperitoneal injection (50 mg/kg × 3). n = 7-10 mice. Statistics were determined by Log-rank test for groups with proportional hazards and Renyi statistics for groups with crossing hazards. p values were adjusted by Bonferroni correction. Source data are provided as a Source Data file.

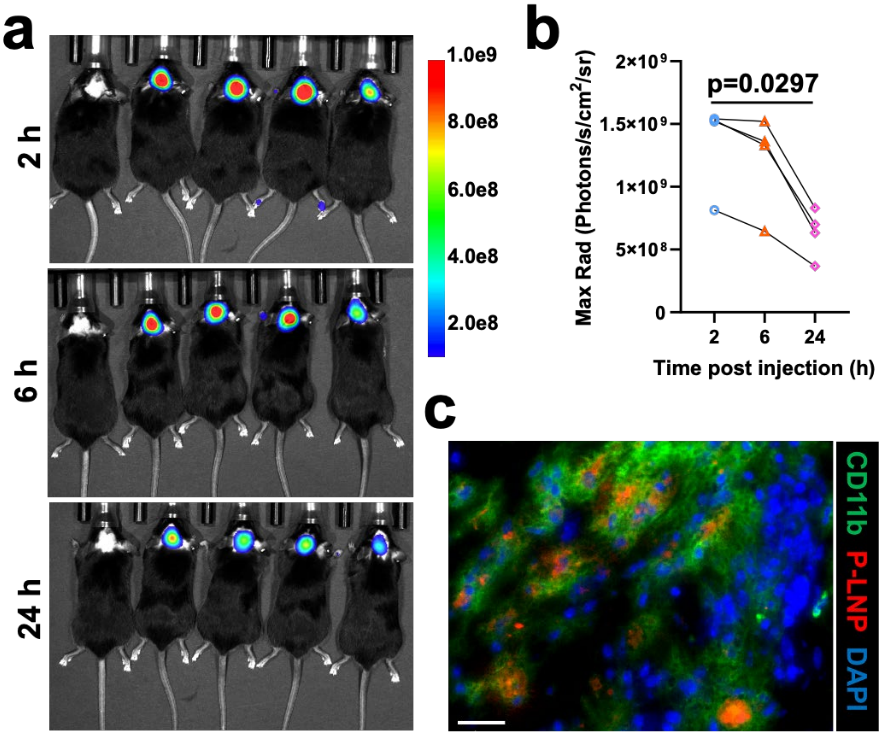

Supplementary Fig. 18 Systemically delivered nanoparticles target TAMCs in brain tumors. C57 mice received intracranial implantation of 7.5×10^4 CT-2A cells, radiotherapy (RT), and one administration of Cy5.5-tagged P-LNP through intravenous injection. Brain distribution of nanoparticles were imaged by live animal imaging (**a**) and quantified by Aura Imaging Software (**b**). The heads of mice were shaved to enable the penetration of fluorescence. n = 4 mice. One mouse without injection was used as background control (left). Statistics were determined by one-way ANOVA with Tukey's multiple comparisons test. **c,** Co-localization of P-LNP and TAMC was determined 24 h post-injection by immunofluorescence staining with anti-mouse/human CD11b Alexa Fluor 488 at 1:100 dilution. The experiment was carried out independently three times. Scale bar, 25 µm. Source data are provided as a Source Data file.

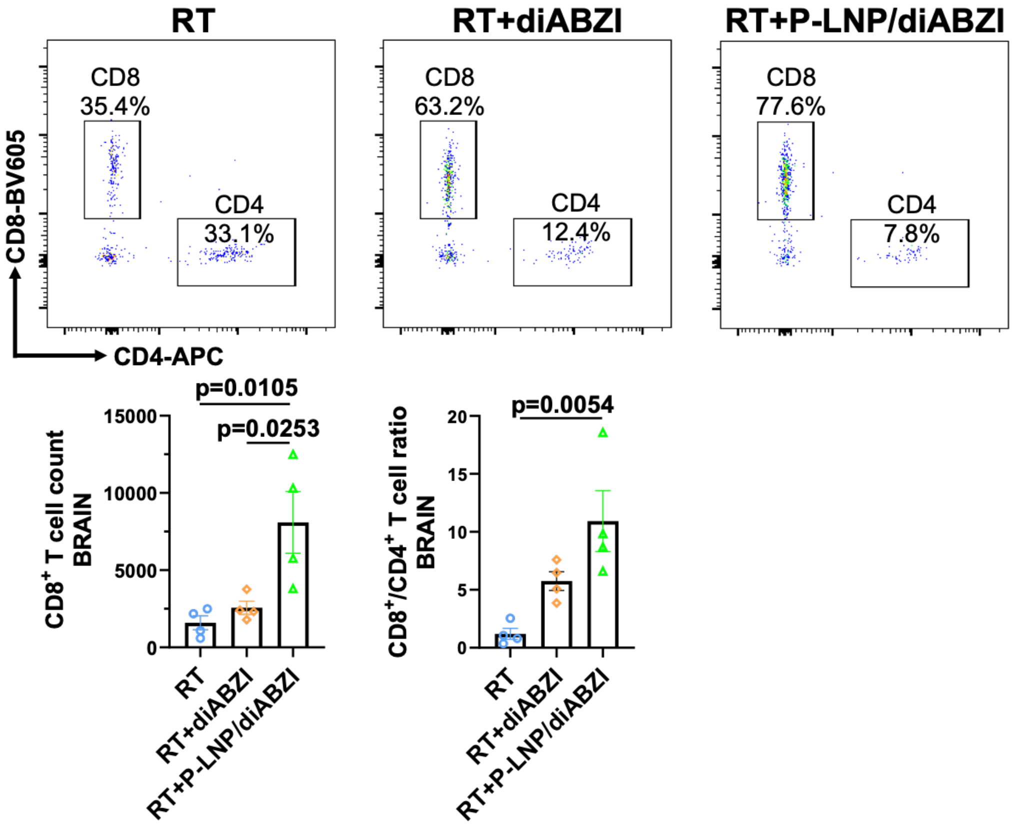

Supplementary Fig. 19 Systemic nanoparticle delivery of diABZI enhances T cell infiltration to brain tumors. C57 mice received intracranial implantation of 7.5 × 10^4 CT-2A cells, radiotherapy (RT, 3 Gy × 3), and two administrations of saline, free diABZI, or P-LNP/diABZI (2.5 mg/kg diABZI) through intravenous injections. Freshly dissected brains were analyzed by flow cytometry for T cell infiltration. n = 4 mice. Statistics were determined by one-way ANOVA with Tukey's multiple comparisons test; data are presented as mean values +/- SEM. Source data are provided as a Source Data file.

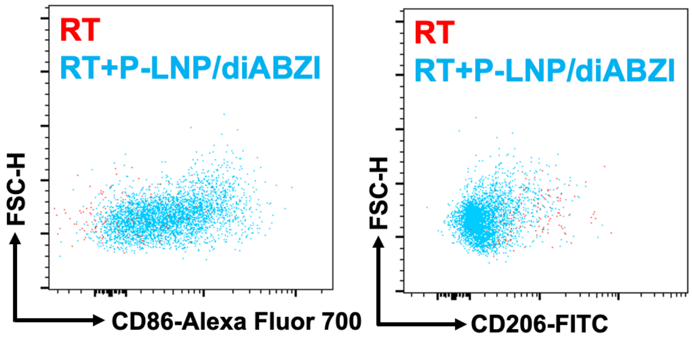

Supplementary Fig. 20 P-LNP/diABZI reprograms TAMC in PVPF8 murine gliomas. Representative dot plots of flow cytometric analysis of CD86 and CD206 expression in TAMCs isolated from C57 mice received intracranial implantation of 7.5×10^4 PVPF8 cells, radiotherapy (RT, 3 Gy × 3), and two administrations of saline or P-LNP/diABZI (2.5 mg/kg diABZI) through intravenous injections. Quantification of mean fluorescence intensity (MFI) was provided in **Fig. 10b**.

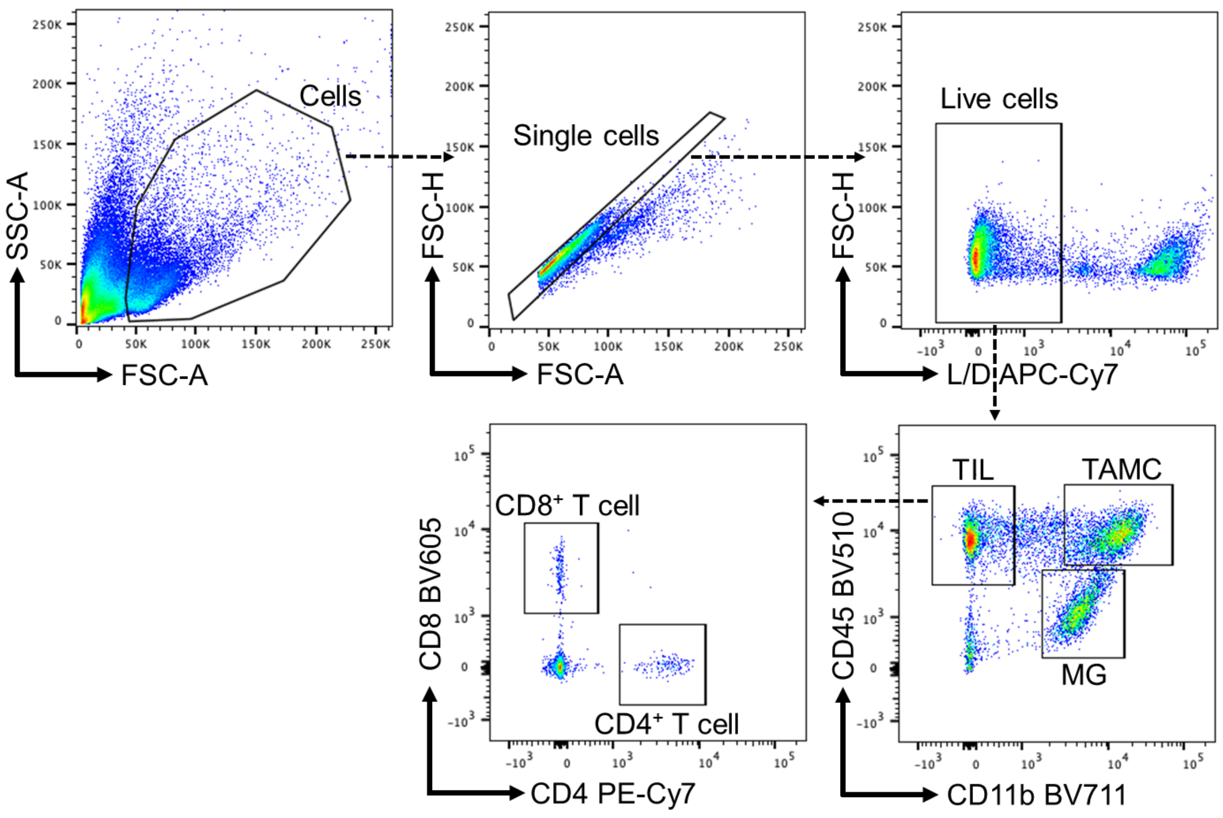

Supplementary Fig. 21 Exemplifying flow cytometry gating strategy for immune profiling of glioma microenvironment. Gating strategy corresponds to flow cytometric analysis in **Fig. 2, 3, 6, 7, 8, 9,** and **10**; and **Supplementary Fig. 1, 5, 6, 7, 8, 12, 13, 14, 19,** and **20**. TAMC, tumor-associated myeloid cell; TIL, tumor-infiltrating lymphocyte; MG, microglia.

Supplementary Table 1 Primer sequences

Primer	5' to 3'
mACTIN (F)	TTG CTG ACA GGA TGC AGA AG
mACTIN (R)	ACA TCT GCT GGA AGG TGG AC
IFNβ (F)	CAG CTC CAA GAA AGG ACG AAC
IFNβ (R)	GGC AGT GTA ACT CTT CTG CAT
IFNα (F)	GGA TGT GAC CTT CCT CAG ACT C
IFNα (R)	ACC TTC TCC TGC GGG AAT CCA A
TNFα (F)	CTG AAC TTC GGG GTG ATC GG
TNFα (R)	GGC TTG TCA CTC GAA TTT TGA GA
CCL5 (F)	GCT GCT TTG CCT ACC TCT CC
CCL5 (R)	TCG AGT GAC AAA CAC GAC TGC
CXCL10 (F)	CCA AGT GCT GCC GTC ATT TTC
CXCL10 (R)	GGC TCG CAG GGA TGA TTT CAA
CXCL11 (F)	GGC TTC CTT ATG TTC AAA CAG GG
CXCL11 (R)	GCC GTT ACT CGG GTA AAT TAC A
IL6 (F)	CCA AGA GGT GAG TGC TTC CC
IL6 (R)	CTG TTG TTC AGA CTC TCT CCC T

Supplementary Table 2 Antibody information

Name	Clone	Vendor	Catalog#	Dilution
anti-human CD45 BV510	2D1	BioLegend	368526	1:50
anti-mouse/human CD11b PE	M1/70	BioLegend	101208	1:50
anti-mouse/human CD11b Alexa Fluor 488	M1/70	BioLegend	101217	1:100
anti-human CD8a Alexa Fluor 700	HIT8a	BioLegend	300920	1:50
anti-human CD4 FITC	OKT4	BioLegend	317408	1:50
anti-human CD69 PerCP-Cyanine5.5	FN50	BioLegend	310926	1:50
anti-human CD25 APC	BC96	BioLegend	302610	1:50
anti-human CD86 BV711	IT2.2	BioLegend	305440	1:50
anti-human PD-1 BV605	EH12.2H7	BioLegend	329924	1:50
human TruStain FcX	3G8/ FUN-2/10.1	BioLegend	422302	1:50
anti-mouse CD45 BV510	30-F11	BioLegend	103138	1:200
anti-mouse/human CD11b BV711	M1/70	BioLegend	101242	1:200
anti-mouse CD80 BV605	16-10A1	BioLegend	104729	1:200
anti-mouse CD86 Alexa Fluor 700	PO3	BioLegend	105122	1:200
anti-mouse CD40 Pacific Blue	3/23	BioLegend	124626	1:200
anti-mouse CD40 APC	3/23	BioLegend	124611	1:200
anti-mouse CD40 PE	3/23	BioLegend	124610	1:200
anti-mouse CD206 FITC	C068C2	BioLegend	141704	1:200
anti-mouse CD206 PE/Cyanine7	C068C2	BioLegend	141720	1:200
anti-mouse Arginase 1 APC	A1exF5	eBioscience	17-3697-82	1:200
anti-mouse iNOS PE	CXNFT	eBioscience	12-5920-82	1:200
anti-mouse CD11c PerCP-Cyanine5.5	N418	BioLegend	117328	1:200
anti-mouse CD11c FITC	N418	BioLegend	117306	1:200

anti-mouse CD8a BV605	53-6.7	BioLegend	100744	1:200
anti-mouse CD4 PE/Cyanine7	RM4-4	BioLegend	116016	1:200
anti-mouse CD4 BUV395	GK1.5	BD Biosciences	565974	1:200
anti-mouse CD4 BV711	RM4-5	BioLegend	100549	1:200
anti-mouse CD4 APC	RM4-5	BioLegend	100516	1:200
anti-mouse CD44 FITC	IM7	BioLegend	103006	1:200
anti-mouse CD62L PerCP-Cyanine5.5	MEL-14	BioLegend	104432	1:200
anti-mouse CD69 BV421	H1.2F3	BioLegend	104545	1:200
anti-mouse CD25 Alexa Fluor 700	PC61	BioLegend	102024	1:200
anti-mouse CD25 PE	PC61	BioLegend	102008	1:200
anti-mouse PD-1 PE	RMP1-30	BioLegend	109104	1:200
anti-mouse Granzyme B Alexa Fluor 647	GB11	BioLegend	515406	1:200
anti-mouse IFN-γ Alexa Fluor 700	XMG1.2	BioLegend	505824	1:200
anti-mouse LAG-3 APC	CC9B7W	BioLegend	125210	1:200
anti-mouse H-2Kb bound to SIINFEKL PE/Cyanine7	25-D1.16	BioLegend	141608	1:200
anti-mouse CD47 PE	MIAP301	BioLegend	127507	1:200
anti-mouse CD47 Alexa Fluor 647	MIAP301	BioLegend	127509	1:100
anti-mouse/rat/human Calreticulin Alexa Fluor 488	D3E6	Cell Signaling	62304S	1:50
anti-mouse PD-L1 PE	10F.9G2	BioLegend	124308	1:200
anti-mouse PD-L1 APC	10F.9G2	BioLegend	124311	1:200
anti-mouse CD8a PE	53-6.7	BioLegend	100708	1:200
anti-mouse CD45.1 PE/Cyanine7	A20	BioLegend	110730	1:200
anti-mouse CD45.2 APC	104	BioLegend	109814	1:200
Biotin anti-human CD45	2D1	BioLegend	368534	1:10
Biotin anti-mouse CD45	30-F11	BioLegend	103104	1:10

anti-mouse CD16/32	93	BioLegend	101302	1:100
anti-mouse/rat/human CD3	SP7	Abcam	ab16669	1:1000
anti-Ovalbumin	Polyclonal	Novus Biologicals	NB600-922SS	1:500
InVivoMAb anti-mouse PD-L1	10F.9G2	BioXCell	BE0101	See below
InVivoMAb anti-mouse CD47	MIAP301	BioXCell	BE0270	See below
InVivoMAb anti-human PD-L1	29E.2A3	BioXCell	BE0285	See below
InVivoMAb anti-human CD47	B6.H12	BioXCell	BE0019-1	See below

For nanoparticle functionalization, weight ratio of 8.7:1 (total lipids to antibody) was used.

Supplementary Table 3 Characteristics of GBM patient samples

Case	NU02747	NU03136
Age at time of collection	71	63
Sex	Female	Male
Race	White	White
Ethnicity	Non-Hispanic	Non-Hispanic
Final Diagnosis, WHO 2021	Glioblastoma	Glioblastoma
WHO grade	Grade 4	Grade 4
New diagnosis vs. recurrent diagnosis	New	New
Temozolomide Received Prior to Resection	No	No
Radiation Received Prior to Resection	No	No
Temozolomide Received Post Resection	Yes	Yes
Radiation Received Post Resection	Yes	Yes
IDH1 Status	Wild-type	Wild-type
IDH2 Status	Wild-type	Wild-type
ATRX Expression	Retained	Retained
P53 Histology	10-15%	5-10%
P53 NGS status	Mutated	Wild-type
Ki-67 Histology	20%	15-20%
MGMT Promotor Methylation	Positive	Positive
1p/19q Codeletion	Not reported	Negative
CDKN2A/B (p16)	Loss	Wild-type
TERT Promoter	Mutated	Mutated
NF1	Wild-type	Wild-type
EGFR Status	Wild-type	EGFR VIII
PTEN status	Altered	Altered
Tumor mutation burden (muts/mb)	not reported	3.1
Year of surgery	2021	2022
