# Supplementary material for: STING agonist-loaded, CD47/PD-L1-targeting nanoparticles potentiate antitumor immunity and radiotherapy for glioblastoma
Source: Nat Commun. 2023 Mar 23;14:1610. doi: 10.1038/s41467-023-37328-9 (PMC10036562; doi:10.1038/s41467-023-37328-9)
Supplement: Supplementary file 3 — Reporting Summary [file 41467_2023_37328_MOESM3_ESM.pdf]

nature portfolio

Corresponding author(s): Maciej Lesniak, Peng Zhang, Jason Miska

Last updated by author(s): 02-22-2023

Reporting Summary

Nature Portfolio wishes to improve the reproducibility of the work that we publish. This form provides structure for consistency and transparency in reporting. For further information on Nature Portfolio policies, see our Editorial Policies and the Editorial Policy Checklist.

Statistics

For all statistical analyses, confirm that the following items are present in the figure legend, table legend, main text, or Methods section.

n/a	Confirmed	
☐	☒	The exact sample size (*n*) for each experimental group/condition, given as a discrete number and unit of measurement
☐	☒	A statement on whether measurements were taken from distinct samples or whether the same sample was measured repeatedly
☐	☒	The statistical test(s) used AND whether they are one- or two-sided<br>*Only common tests should be described solely by name; describe more complex techniques in the Methods section.*
☒	☐	A description of all covariates tested
☐	☒	A description of any assumptions or corrections, such as tests of normality and adjustment for multiple comparisons
☐	☒	A full description of the statistical parameters including central tendency (e.g. means) or other basic estimates (e.g. regression coefficient) AND variation (e.g. standard deviation) or associated estimates of uncertainty (e.g. confidence intervals)
☐	☒	For null hypothesis testing, the test statistic (e.g. *F*, *t*, *r*) with confidence intervals, effect sizes, degrees of freedom and *P* value noted<br>*Give P values as exact values whenever suitable.*
☒	☐	For Bayesian analysis, information on the choice of priors and Markov chain Monte Carlo settings
☒	☐	For hierarchical and complex designs, identification of the appropriate level for tests and full reporting of outcomes
☒	☐	Estimates of effect sizes (e.g. Cohen's *d*, Pearson's *r*), indicating how they were calculated

Our web collection on statistics for biologists contains articles on many of the points above.

Software and code

Policy information about availability of computer code

Data collection	Flow cytometry data were collected using FACS DIVA 8.0.3 software. Metabolomics data were collected using Xclibur 4.1 software. qPCR data were collected using Bio-Rad CFX Manager 3.1. Microscopy images were collected using Leica Application Suite X 2.0. Size distribution and zeta-potential data were collected using Malvern Zetasizer Software 8.01. Incucyte data were collected using Sartorius IncuCyte 2020A. Histology images were collected using NDP view2 on a Nanozoomer 2.0HT. Live animal imaging was acquired by Aura imaging software. scRNA-seq data were acquired using CellRanger 6.0 software on 10X Genomics Chromium controller. Western Blot images were collected using Bio-Rad Image Lab 6.1 Software. Mass Spectrum data was collected on the Bruker Amazon X using HyStar Ver 3.2 data acquisition software.
Data analysis	GraphPad Prism 9.5 was used for statistical analysis. Flow cytometry data were analyzed using FlowJo 10.6.1 software. Metabolite analysis was performed by Tracefinder 4.1 software and Metaboanalyst software. Mass Spectrum data were processed using QuantAnalysis Ver 4.4 for data analysis. scRNA-seq data were processed using Python 3.8, Scanpy 1.9. Cell type annotation was performed using mouse RNA-seq reference and R package SingleR. R package AUCell was used to calculate enrichment score for each of the gene sets. Gene sets were obtained from MSigDB v7.1, utilizing the C5: GO gene sets collection. Pathway analysis was performed using Metascape. Incucyte data were analyzed using Sartorius IncuCyte 2020A. Histology images were processed and analyzed using NDP.view 2.8 and ImageJ 2.3.0/1.53q/ Java 1.8.0_66 (64 bit). Live animal imaging was processed by Aura imaging software 2.3.1.

For manuscripts utilizing custom algorithms or software that are central to the research but not yet described in published literature, software must be made available to editors and reviewers. We strongly encourage code deposition in a community repository (e.g. GitHub). See the Nature Portfolio guidelines for submitting code & software for further information.

Data

Policy information about availability of data

All manuscripts must include a data availability statement. This statement should provide the following information, where applicable:

- Accession codes, unique identifiers, or web links for publicly available datasets
- A description of any restrictions on data availability
- For clinical datasets or third party data, please ensure that the statement adheres to our policy

The Single-cell RNA sequencing data files have been deposited in the NCBI Sequence Read Archive (SRA) database under the accession number PRJNA903231 (https://urldefense.com/v3/__https://www.ncbi.nlm.nih.gov/sra/PRJNA903231__;!!Dq0X2DkFhyF93HkjWTBQKhk!Xu_fgAX0GO7hjvNEe8-3MKFTNb2RK-C7MAQ3JHiqjDsGpBDObEqVXlRJMsPzAy8JhH_IRWf3VJn5IOguPjPtZw$). The metabolomics data have been deposited in the Metabolomics Workbench under the Project ID PR001593 (http://dx.doi.org/10.21228/M8SH9M). Source data are available in the source data file. Source data are provided with this paper.

Human research participants

Policy information about studies involving human research participants and Sex and Gender in Research.

Reporting on sex and gender	This study was not sex/gender-specific. Both genders were included.
Population characteristics	Detailed patient sample information was provided as a Supplementary Table.
Recruitment	Patients presenting for a neurosurgical procedure for the treatment of a nervous system tumor and meet the inclusion criteria will be consented for participation in the tumor tissue bank at the time of consent for the surgical procedure.
Ethics oversight	IRB number: STU00095863, STU00202003

Note that full information on the approval of the study protocol must also be provided in the manuscript.

Field-specific reporting

Please select the one below that is the best fit for your research. If you are not sure, read the appropriate sections before making your selection.

☒ Life sciences ☐ Behavioural & social sciences ☐ Ecological, evolutionary & environmental sciences

For a reference copy of the document with all sections, see nature.com/documents/nr-reporting-summary-flat.pdf

Life sciences study design

All studies must disclose on these points even when the disclosure is negative.

Sample size	Sample size was approximated based on previous studies, pilot experiments, and standard protocols in the field to provide sufficient power for statistical analysis without predetermination. For power analysis, our preliminary data showed that the effect size of treatments (%) compared with controls is 6.96. Therefore, with the sample size of 3 per group, we have the power of over 0.95 at an alpha level of 0.01 to detect the effect (Calculated from Miska et al. 2021, Science Advances). $n \geq 3$ was used for all experiments to ensure the reproducibility. Detailed sample size information is noted in the figure legends.
Data exclusions	Cells of poor quality in scRNA-seq samples were excluded if cells with (i) <200 detected genes, (ii) >40000 outlier number of UMIs, and (iii) proportion of mitochondrial gene expression >20%.
Replication	At least three replicates were analyzed in each independent experiment to ensure the experimental results were reliable. Biological and technical replicates were considered. At least 2 or 3 independent experiments were performed to validate key data. Multiple assays, such as scRNA-seq analysis, metabolomic analysis, and flow cytometry analysis, were performed to cross-validate key findings.
Randomization	Experimental animals and samples were andomly assigned to into different treatment groups.
Blinding	Researchers were not blinded due to the workforce or experimental design. Critical experiments were analyzed by at least 2 independent researchers for unbiased conclusion.

Reporting for specific materials, systems and methods

We require information from authors about some types of materials, experimental systems and methods used in many studies. Here, indicate whether each material, system or method listed is relevant to your study. If you are not sure if a list item applies to your research, read the appropriate section before selecting a response.

Materials & experimental systems

n/a	Involved in the study	
	☒	Antibodies
	☒	Eukaryotic cell lines
☒		Palaeontology and archaeology
	☒	Animals and other organisms
☒		Clinical data
☒		Dual use research of concern

Methods

n/a	Involved in the study	
☒		ChIP-seq
	☒	Flow cytometry
☒		MRI-based neuroimaging

Antibodies

Antibodies used

anti-human CD45 BV510, Clone 2D1, BioLegend, Catalog# 368526, RRID# AB_2687377
anti-mouse/human CD11b PE, Clone M1/70, BioLegend, Catalog#101208, RRID# AB_312791
anti-mouse/human CD11b Alexa Fluor 488, BioLegend, Catalog#101217, RRID# AB_389305
anti-human CD8a Alexa Fluor 700, Clone HIT8a, BioLegend, Catalog# 300920, RRID# AB_528885
anti-human CD4 FITC, Clone OKT4, BioLegend, Catalog# 317408, RRID# AB_571951
anti-human CD69 PerCP/Cyanine5.5, Clone FN50, BioLegend, Catalog# 310926, RRID# AB_2074956
anti-human CD25 APC, Clone BC96, BioLegend, Catalog# 302610, RRID# AB_314280
anti-human CD86 BV711, Clone IT2.2, BioLegend, Catalog# 305440, RRID# AB_2565835
anti-human PD-1 BV605, Clone EH12.2H7, BioLegend, Catalog# 329924, RRID# AB_2563212
Human TruStain FcX, CD16 (Clone 3G8), CD32 (Clone FUN-2), CD64 (Clone 10.1), BioLegend, Catalog# 422302, RRID# AB_2818986
anti-mouse CD45 BV510, Clone 30-F11, BioLegend, Catalog# 103138, RRID# AB_2563061
anti-mouse/human CD11b BV711, Clone M1/70, BioLegend, Catalog# 101242, RRID# AB_2563310
anti-mouse CD80 BV605, Clone 16-10A1, BioLegend, Catalog# 104729, RRID# AB_11126141
anti-mouse CD86 Alexa Fluor 700, Clone PO3, BioLegend, Catalog# 105122, RRID# AB_493723
anti-mouse CD40 Pacific Blue, Clone 3/23, BioLegend, Catalog# 124626, RRID# AB_2561476
anti-mouse CD40 APC, Clone 3/23, BioLegend, Catalog# 124611, RRID# AB_1134081
anti-mouse CD40 PE, Clone 3/23, BioLegend, Catalog# 124610, RRID# AB_1134075
anti-mouse CD206 FITC, Clone C068C2, BioLegend, Catalog# 141704, RRID# AB_10901166
anti-mouse CD206 PE/Cyanine7, Clone C068C2, BioLegend, Catalog# 141720, RRID# AB_2562248
anti-mouse Arginase 1 APC, Clone A1exF5, eBioscience, Catalog# 17-3697-82, RRID# AB_2734835
anti-mouse iNOS PE, Clone CXNFT, eBioscience, Catalog# 12-5920-82, RRID# AB_2572642
anti-mouse CD11c PerCP/Cyanine5.5, Clone N418, BioLegend, Catalog# 117328, RRID# AB_2129641
anti-mouse CD11c FITC, Clone N418, BioLegend, Catalog# 117306, RRID# AB_313775
anti-mouse CD8a BV605, Clone 53-6.7, BioLegend, Catalog# 100744, RRID# AB_2562609
anti-mouse CD4 PE/Cyanine7, Clone RM4-4, BioLegend, Catalog# 116016, RRID# AB_2563111
anti-mouse CD4 BUV395, Clone GK1.5, BD Biosciences, Catalog# 565974, RRID# AB_2738426
anti-mouse CD44 FITC, Clone IM7, BioLegend, Catalog# 103006, RRID# AB_312957
anti-mouse CD62L PerCP/Cyanine5.5, Clone MEL-14, BioLegend, Catalog# 104432, RRID# AB_2285839
anti-mouse CD69 BV421, Clone H1.2F3, BioLegend, Catalog# 104545, RRID# AB_2686969
anti-mouse CD25 Alexa Fluor 700, Clone PC61, BioLegend, Catalog# 102024, RRID# AB_493709
anti-mouse CD25 PE, Clone PC61, BioLegend, Catalog# 102008, RRID# AB_312857
anti-mouse PD1 PE, Clone RMP1-30, BioLegend, Catalog# 109104, RRID# AB_31342
anti-mouse Granzyme B Alexa Fluor 647, Clone GB11, BioLegend, Catalog# 515406, RRID# AB_2566333
anti-mouse IFN-γ Alexa Fluor 700, Clone XMG1.2, BioLegend, Catalog# 505824, RRID# AB_2561300
anti-mouse LAG-3 APC, Clone CC9B7W, BioLegend, Catalog# 125210, RRID# AB_10639727
anti-mouse H-2Kb bound to SIINFEKL PE/Cyanine7, Clone 25-D1.16, BioLegend, Catalog# 141608, RRID# AB_11218593
anti-mouse CD47 PE, Clone miap301, BioLegend, Catalog# 127507, RRID# AB_1134133
anti-mouse CD47 Alexa Fluor 647, clone miap301, BioLegend, Catalog# 127509, RRID# AB_1134121
anti-mouse/rat/human Calreticulin, Alexa Fluor 488, Clone D3E6, Cell Signaling, Catalog# 62304S
anti-mouse PD-L1 APC, Clone 10F.9G2, BioLegend, Catalog# 124311, RRID# AB_10612935
anti-mouse CD8a PE, Clone 53-6.7, BioLegend, Catalog# 100708, RRID# AB_312747
anti-mouse CD45.1 PE/Cyanine7, Clone A20, BioLegend, Catalog# 110730, RRID# AB_1134168
anti-mouse CD45.2 APC, Clone 104, BioLegend, Catalog# 109814, RRID# AB_389211
Biotin anti-human CD45 Antibody, Clone 2D1, BioLegend, Catalog# 368534, RRID# AB_2721498
Biotin anti-mouse CD45 Antibody, Clone 30-F11, BioLegend, Catalog# 103104, RRID# AB_312969
Anti-mouse CD16/32 Antibody, Clone 93 BioLegend, Catalog#, 101302, RRID# AB_312801
InVivoMAb anti-mouse PD-L1, Clone 10F.9G2, BioXCell, Catalog# BE0101, RRID# AB_10949073
InVivoMAb anti-mouse CD47, Clone MIAP301, BioXCell, Catalog# BE0270, RRID# AB_2687793
InVivoMAb anti-human PD-L1, Clone 29E.2A3, BioXCell, Catalog# BE0285, RRID# AB_2687808
InVivoMAb anti-human CD47, Clone B6.H12, BioXCell, Catalog# BE0019-1, RRID# AB_1107655
Ovalbumin antibody, Polyclonal, Novus Biologicals, Catalog# NB600-922SS

Validation

All listed primary antibodies (anti-mouse and anti-human) for flow cytometry, blocking, immunofluorescence staining, and Western blot applications were well-established and commercialized products that have been extensively tested and validated by the

manufacturers as stated on the websites (BioLegend, BioXCell, eBioscience, Cell Signaling, and Novus Biologicals.

Eukaryotic cell lines

Policy information about cell lines and Sex and Gender in Research

Cell line source(s)	CT-2A murine glioma cells were obtained as a gift from Dr. Tom Seyfried at Boston College. PVPF8 murine glioma cells were originally generated by and obtained from Dr. Adam Sonabend at Northwestern University.
Authentication	Cell lines were authenticated by identification of short tandem repeat (STR) markers.
Mycoplasma contamination	Cell lines were tested negative for mycoplasma contamination.
Commonly misidentified lines (See ICLAC register)	No commonly misidentified cell lines were used.

Animals and other research organisms

Policy information about studies involving animals; ARRIVE guidelines recommended for reporting animal research, and Sex and Gender in Research

Laboratory animals	C57BL/6, CD45.1 C57BL/6, Rag1 deficient, OT-I, and STINGgt mice were all purchased from the Jackson Laboratory. Animals were bred and housed in a standard barrier animal facility at Northwestern University with the light cycle of 14:10, ambient temperature at 22 °C, and relative humidity range between 30-70%. Experimental animals were mixed-gender and randomly assigned to into different treatment groups at 6 to 8 weeks old. Experimental and control animals were co-housed. All animal-related experiments were performed in full compliance with animal protocols approved by the Northwestern University Institutional Animal Care and Use Committee (IACUC).
Wild animals	No wild animals were included.
Reporting on sex	Experimental animals were mixed-gender, age-matched, and randomly assigned to into different treatment groups.
Field-collected samples	No field-collected samples were used.
Ethics oversight	All animal-related experiments were performed in full compliance with animal protocols approved by the Northwestern University Institutional Animal Care and Use Committee (IACUC).

Note that full information on the approval of the study protocol must also be provided in the manuscript.

Flow Cytometry

Plots

Confirm that:

- [x] The axis labels state the marker and fluorochrome used (e.g. CD4-FITC).
- [x] The axis scales are clearly visible. Include numbers along axes only for bottom left plot of group (a 'group' is an analysis of identical markers).
- [x] All plots are contour plots with outliers or pseudocolor plots.
- [x] A numerical value for number of cells or percentage (with statistics) is provided.

Methodology

Sample preparation	Sample preparation is described in detail in Methods of the the manuscript. For murine samples, single cell suspensions were pre-blocked with anti-CD16/32 antibodies before staining. Dead cells were excluded using the Fixable Viability Dye eFluor780. The Foxp3 fixation/permeabilization protocol was used for intracellular staining. For cytokine staining, cells were pre-incubated with cell stimulation cocktail plus protein transport inhibitors for 4 h. All antibodies were used at a 1:200 dilution except as otherwise specified. For human samples, all antibodies were used at 1:50 dilution. Cells were pre-blocked with Human TruStain FcX for 15 min at 4 °C before antibody staining.
Instrument	BD FACSymphony A5, BD Fortessa LSRII
Software	Flow cytometry data were collected using FACS DIVA 8.0.3 software and analyzed using FlowJo 10.6.1 software.
Cell population abundance	>97% post-sorting as determined by flow cytometry analysis.

Gating strategy

FSC-A vs SSC-A plot was used to determine cell populations from debris, followed by FSC-A vs FSC-H to determine singlets. Live cells were then gated based on viability dye staining. Myeloid and lymphoid compartments were gated as CD45+CD11b+ and CD45+ CD11b- populations, respectively. CD8+ and CD4+ T cells were further gated in lymphoid population based on CD8 + and CD4+ staining. Percentage of positive cells and/or MFI were used to measure expression of target markers. Further gating strategy is provided as a Supplementery Figure.

☒ Tick this box to confirm that a figure exemplifying the gating strategy is provided in the Supplementary Information.
