# Supplementary material for: STING agonist-loaded, CD47/PD-L1-targeting nanoparticles potentiate antitumor immunity and radiotherapy for glioblastoma
Source: Nat Commun. 2023 Mar 23;14:1610. doi: 10.1038/s41467-023-37328-9 (PMC10036562; doi:10.1038/s41467-023-37328-9)
Supplement: Supplementary file 4 — Description of Additional Supplementary Files [file 41467_2023_37328_MOESM4_ESM.docx]

**Description of Additional Supplementary Files**

Supplementary Data 1

Description: Single-cell RNA sequencing analysis of genes that are upregulated in different TAMC sub-clusters.

Supplementary Data 2

Description: Single-cell RNA sequencing analysis of genes that are upregulated in TAMCs post-radiotherapy or combination therapy.

Supplementary Data 3

Description: Single-cell RNA sequencing analysis of genes that are upregulated in T cells post-radiotherapy or combination therapy.
